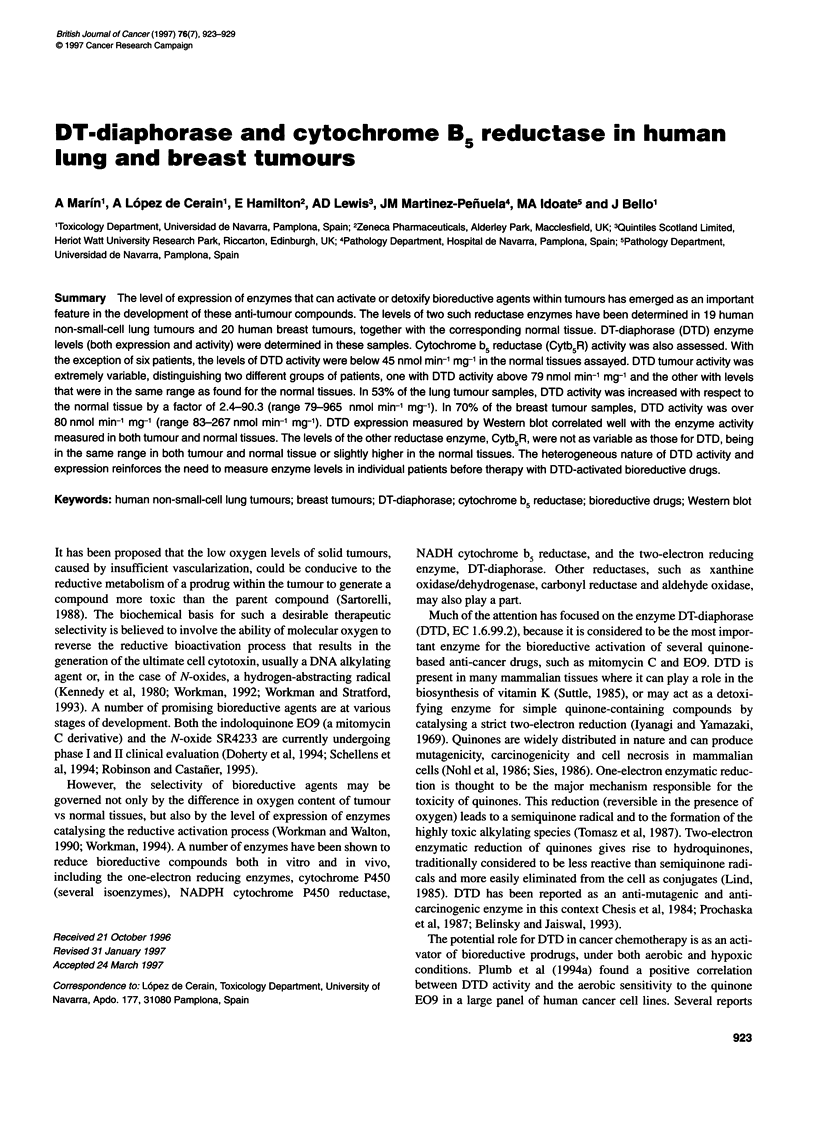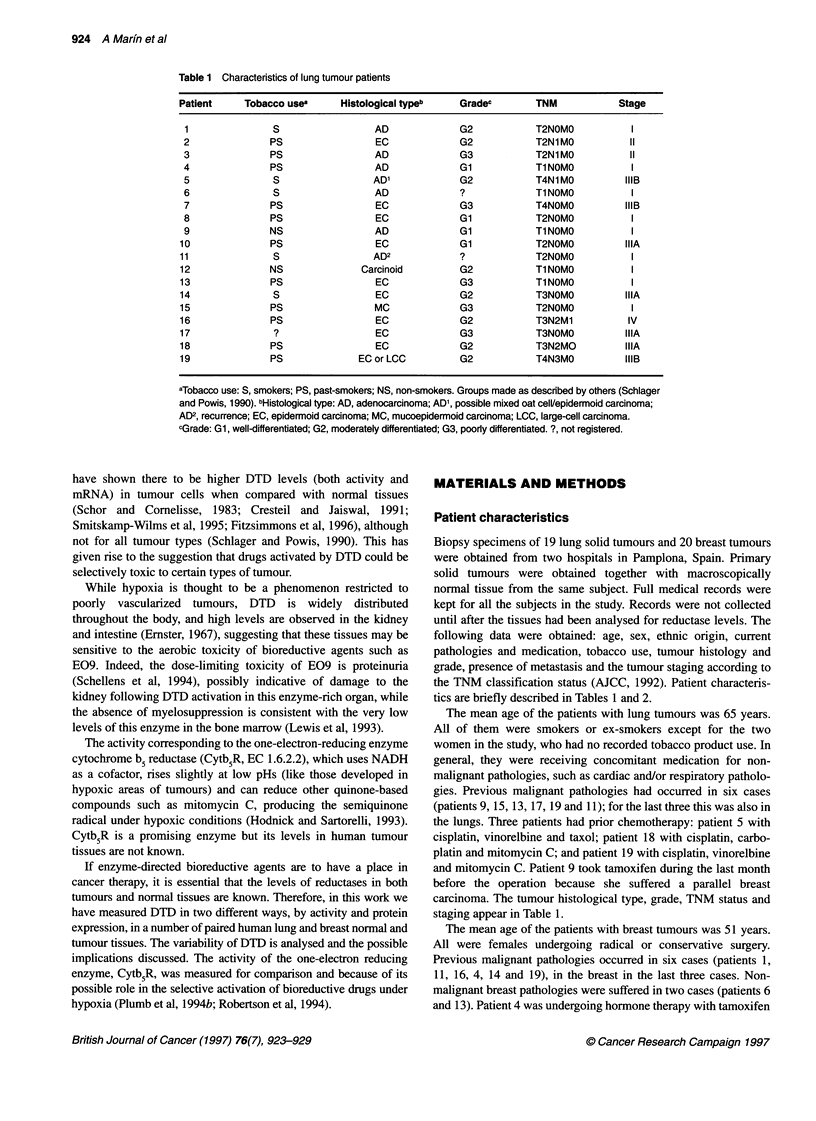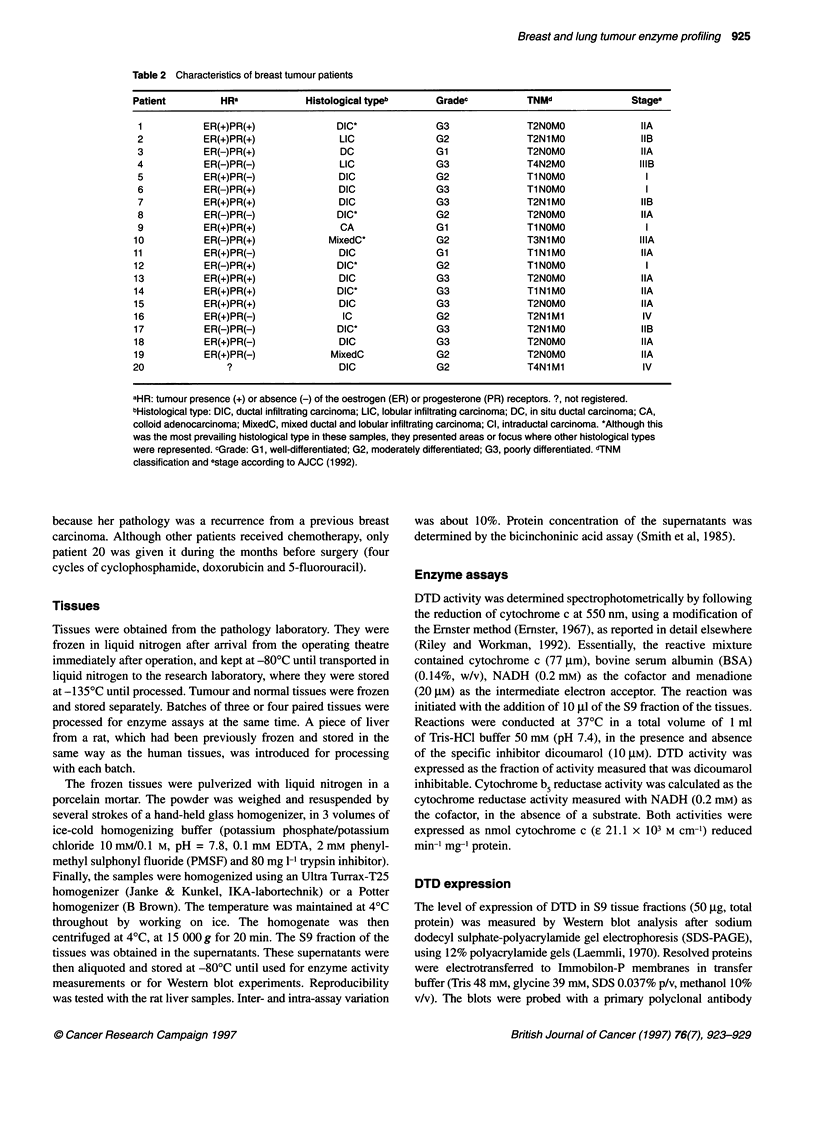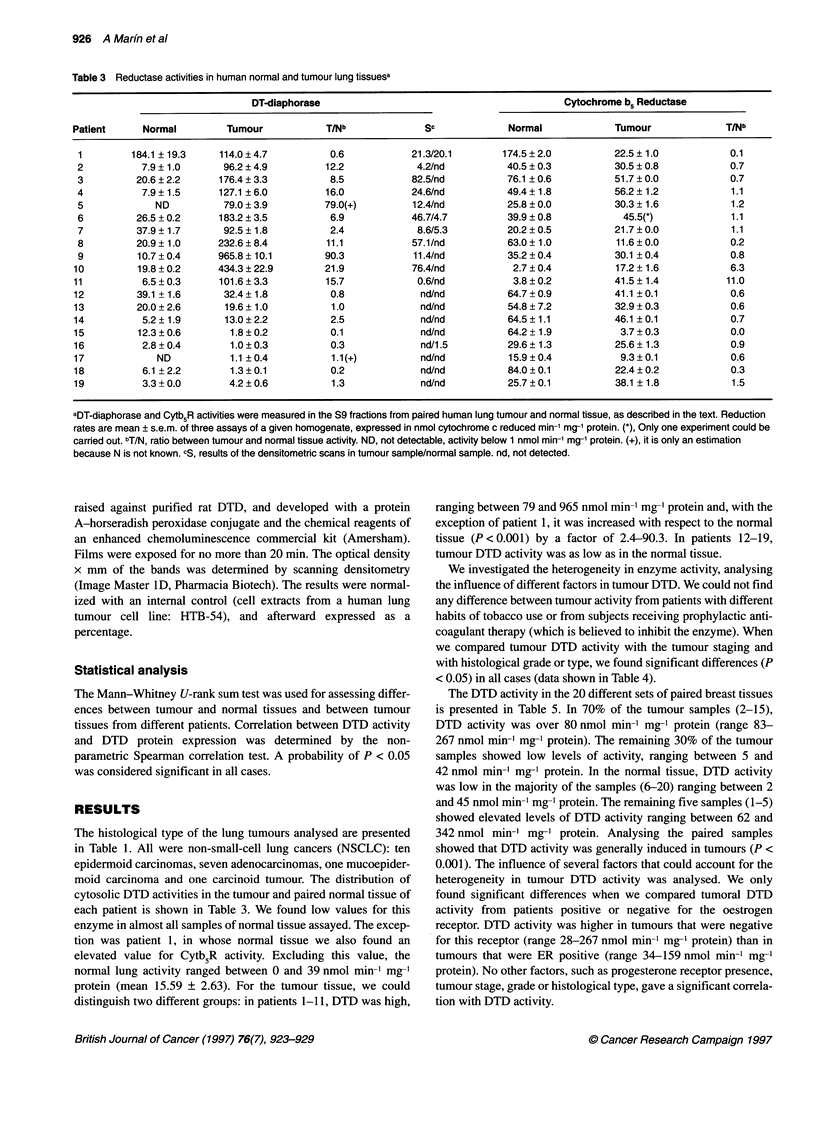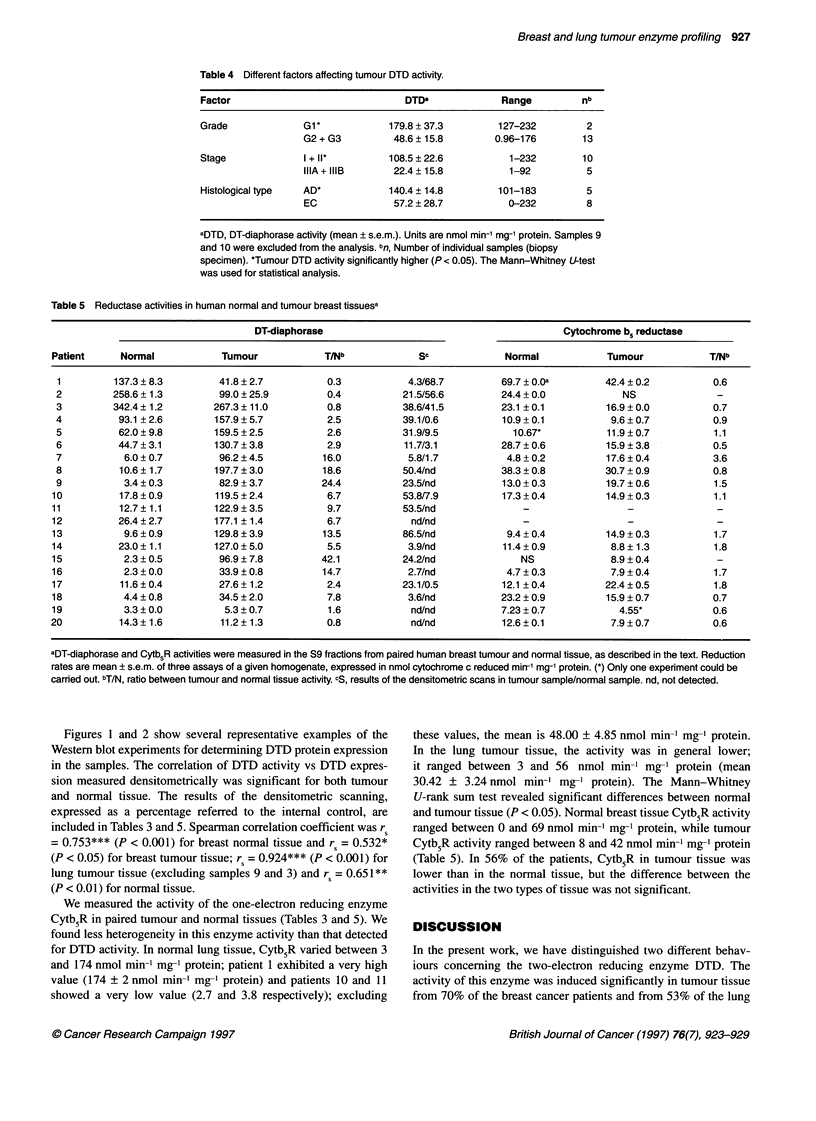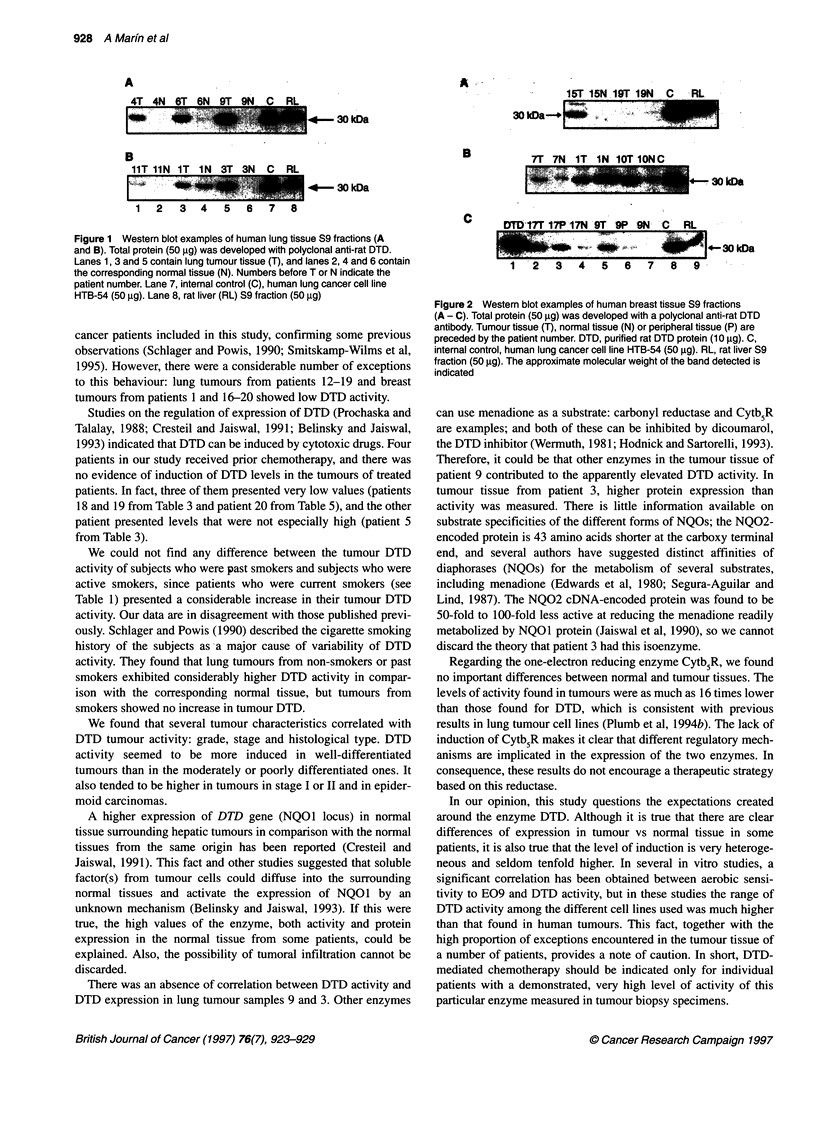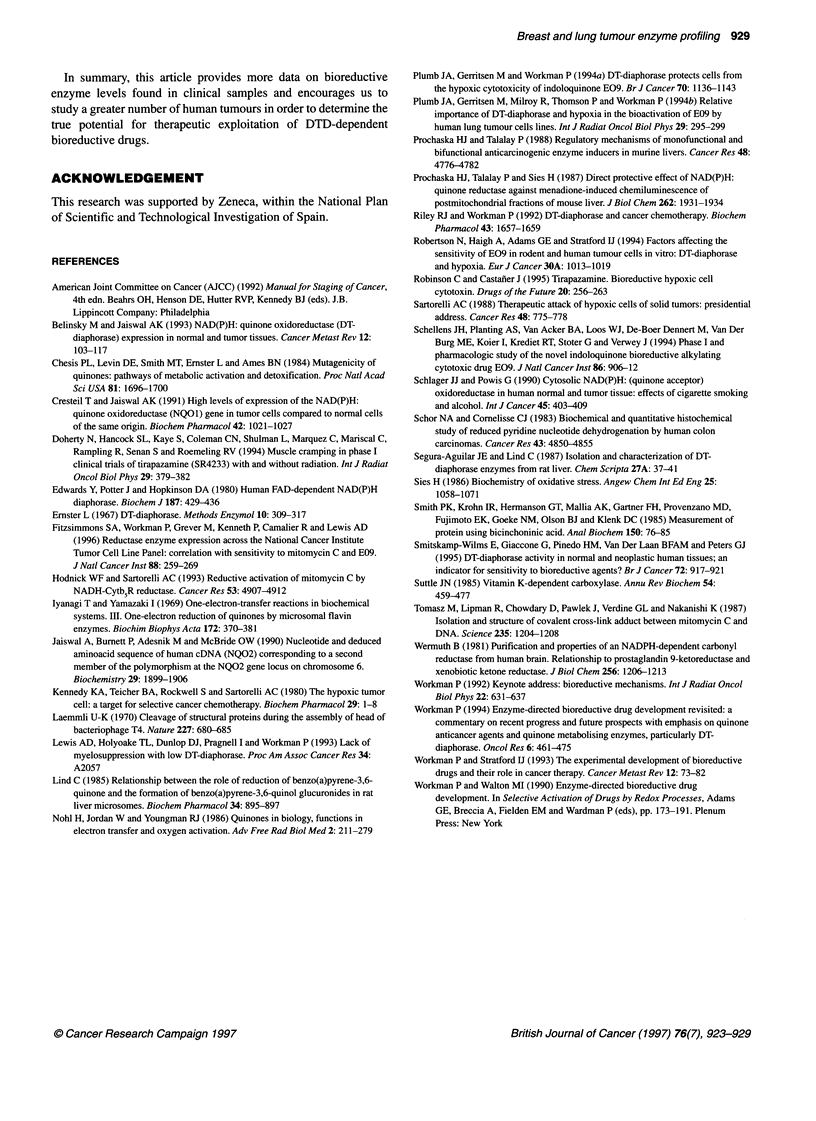# DT-diaphorase and cytochrome B5 reductase in human lung and breast tumours.

**DOI:** 10.1038/bjc.1997.485

**Published:** 1997

**Authors:** A. MarÃ­n, A. LÃ³pez de Cerain, E. Hamilton, A. D. Lewis, J. M. Martinez-PeÃ±uela, M. A. Idoate, J. Bello

**Affiliations:** Toxicology Department, Universidad de Navarra, Pamplona, Spain.

## Abstract

**Images:**


British Journal of Cancer (1997) 76(7), 923–929
© 1997 Cancer Research Campaign

### DT-diaphorase and cytochrome $B_5$ reductase in human lung and breast tumours

A Marín[1], A López de Cerain[1], E Hamilton[2], AD Lewis[3], JM Martinez-Peñuela[4], MA Idoate[5] and J Bello[1]

[1]Toxicology Department, Universidad de Navarra, Pamplona, Spain; [2]Zeneca Pharmaceuticals, Alderley Park, Macclesfield, UK; [3]Quintiles Scotland Limited, Heriot Watt University Research Park, Riccarton, Edinburgh, UK; [4]Pathology Department, Hospital de Navarra, Pamplona, Spain; [5]Pathology Department, Universidad de Navarra, Pamplona, Spain

**Summary** The level of expression of enzymes that can activate or detoxify bioreductive agents within tumours has emerged as an important feature in the development of these anti-tumour compounds. The levels of two such reductase enzymes have been determined in 19 human non-small-cell lung tumours and 20 human breast tumours, together with the corresponding normal tissue. DT-diaphorase (DTD) enzyme levels (both expression and activity) were determined in these samples. Cytochrome $b_5$ reductase ($Cytb_5R$) activity was also assessed. With the exception of six patients, the levels of DTD activity were below 45 nmol $min^{-1}$ $mg^{-1}$ in the normal tissues assayed. DTD tumour activity was extremely variable, distinguishing two different groups of patients, one with DTD activity above 79 nmol $min^{-1}$ $mg^{-1}$ and the other with levels that were in the same range as found for the normal tissues. In 53% of the lung tumour samples, DTD activity was increased with respect to the normal tissue by a factor of 2.4–90.3 (range 79–965 nmol $min^{-1}$ $mg^{-1}$). In 70% of the breast tumour samples, DTD activity was over 80 nmol $min^{-1}$ $mg^{-1}$ (range 83–267 nmol $min^{-1}$ $mg^{-1}$). DTD expression measured by Western blot correlated well with the enzyme activity measured in both tumour and normal tissues. The levels of the other reductase enzyme, $Cytb_5R$, were not as variable as those for DTD, being in the same range in both tumour and normal tissue or slightly higher in the normal tissues. The heterogeneous nature of DTD activity and expression reinforces the need to measure enzyme levels in individual patients before therapy with DTD-activated bioreductive drugs.

**Keywords:** human non-small-cell lung tumours; breast tumours; DT-diaphorase; cytochrome $b_5$ reductase; bioreductive drugs; Western blot

It has been proposed that the low oxygen levels of solid tumours, caused by insufficient vascularization, could be conducive to the reductive metabolism of a prodrug within the tumour to generate a compound more toxic than the parent compound (Sartorelli, 1988). The biochemical basis for such a desirable therapeutic selectivity is believed to involve the ability of molecular oxygen to reverse the reductive bioactivation process that results in the generation of the ultimate cell cytotoxin, usually a DNA alkylating agent or, in the case of *N*-oxides, a hydrogen-abstracting radical (Kennedy et al, 1980; Workman, 1992; Workman and Stratford, 1993). A number of promising bioreductive agents are at various stages of development. Both the indoloquinone EO9 (a mitomycin C derivative) and the *N*-oxide SR4233 are currently undergoing phase I and II clinical evaluation (Doherty et al, 1994; Schellens et al, 1994; Robinson and Castañer, 1995).

However, the selectivity of bioreductive agents may be governed not only by the difference in oxygen content of tumour vs normal tissues, but also by the level of expression of enzymes catalysing the reductive activation process (Workman and Walton, 1990; Workman, 1994). A number of enzymes have been shown to reduce bioreductive compounds both in vitro and in vivo, including the one-electron reducing enzymes, cytochrome P450 (several isoenzymes), NADPH cytochrome P450 reductase, NADH cytochrome $b_5$ reductase, and the two-electron reducing enzyme, DT-diaphorase. Other reductases, such as xanthine oxidase/dehydrogenase, carbonyl reductase and aldehyde oxidase, may also play a part.

Much of the attention has focused on the enzyme DT-diaphorase (DTD, EC 1.6.99.2), because it is considered to be the most important enzyme for the bioreductive activation of several quinone-based anti-cancer drugs, such as mitomycin C and EO9. DTD is present in many mammalian tissues where it can play a role in the biosynthesis of vitamin K (Suttle, 1985), or may act as a detoxifying enzyme for simple quinone-containing compounds by catalysing a strict two-electron reduction (Iyanagi and Yamazaki, 1969). Quinones are widely distributed in nature and can produce mutagenicity, carcinogenicity and cell necrosis in mammalian cells (Nohl et al, 1986; Sies, 1986). One-electron enzymatic reduction is thought to be the major mechanism responsible for the toxicity of quinones. This reduction (reversible in the presence of oxygen) leads to a semiquinone radical and to the formation of the highly toxic alkylating species (Tomasz et al, 1987). Two-electron enzymatic reduction of quinones gives rise to hydroquinones, traditionally considered to be less reactive than semiquinone radicals and more easily eliminated from the cell as conjugates (Lind, 1985). DTD has been reported as an anti-mutagenic and anti-carcinogenic enzyme in this context Chesis et al, 1984; Prochaska et al, 1987; Belinsky and Jaiswal, 1993).

The potential role for DTD in cancer chemotherapy is as an activator of bioreductive prodrugs, under both aerobic and hypoxic conditions. Plumb et al (1994a) found a positive correlation between DTD activity and the aerobic sensitivity to the quinone EO9 in a large panel of human cancer cell lines. Several reports

*Received 21 October 1996*
*Revised 31 January 1997*
*Accepted 24 March 1997*

*Correspondence to:* López de Cerain, Toxicology Department, University of Navarra, Apdo. 177, 31080 Pamplona, Spain

Table 1 Characteristics of lung tumour patients

| Patient | Tobacco use[a] | Histological type[b] | Grade[c] | TNM | Stage |
|---|---|---|---|---|---|
| 1 | S | AD | G2 | T2N0M0 | I |
| 2 | PS | EC | G2 | T2N1M0 | II |
| 3 | PS | AD | G3 | T2N1M0 | II |
| 4 | PS | AD | G1 | T1N0M0 | I |
| 5 | S | AD[1] | G2 | T4N1M0 | IIIB |
| 6 | S | AD | ? | T1N0M0 | I |
| 7 | PS | EC | G3 | T4N0M0 | IIIB |
| 8 | PS | EC | G1 | T2N0M0 | I |
| 9 | NS | AD | G1 | T1N0M0 | I |
| 10 | PS | EC | G1 | T2N0M0 | IIIA |
| 11 | S | AD[2] | ? | T2N0M0 | I |
| 12 | NS | Carcinoid | G2 | T1N0M0 | I |
| 13 | PS | EC | G3 | T1N0M0 | I |
| 14 | S | EC | G2 | T3N0M0 | IIIA |
| 15 | PS | MC | G3 | T2N0M0 | I |
| 16 | PS | EC | G2 | T3N2M1 | IV |
| 17 | ? | EC | G3 | T3N0M0 | IIIA |
| 18 | PS | EC | G2 | T3N2MO | IIIA |
| 19 | PS | EC or LCC | G2 | T4N3M0 | IIIB |

[a]Tobacco use: S, smokers; PS, past-smokers; NS, non-smokers. Groups made as described by others (Schlager and Powis, 1990). [b]Histological type: AD, adenocarcinoma; AD[1], possible mixed oat cell/epidermoid carcinoma; AD[2], recurrence; EC, epidermoid carcinoma; MC, mucoepidermoid carcinoma; LCC, large-cell carcinoma. [c]Grade: G1, well-differentiated; G2, moderately differentiated; G3, poorly differentiated. ?, not registered.

have shown there to be higher DTD levels (both activity and mRNA) in tumour cells when compared with normal tissues (Schor and Cornelisse, 1983; Cresteil and Jaiswal, 1991; Smitskamp-Wilms et al, 1995; Fitzsimmons et al, 1996), although not for all tumour types (Schlager and Powis, 1990). This has given rise to the suggestion that drugs activated by DTD could be selectively toxic to certain types of tumour.

While hypoxia is thought to be a phenomenon restricted to poorly vascularized tumours, DTD is widely distributed throughout the body, and high levels are observed in the kidney and intestine (Ernster, 1967), suggesting that these tissues may be sensitive to the aerobic toxicity of bioreductive agents such as EO9. Indeed, the dose-limiting toxicity of EO9 is proteinuria (Schellens et al, 1994), possibly indicative of damage to the kidney following DTD activation in this enzyme-rich organ, while the absence of myelosuppression is consistent with the very low levels of this enzyme in the bone marrow (Lewis et al, 1993).

The activity corresponding to the one-electron-reducing enzyme cytochrome $b_5$ reductase (Cyt$b_5$R, EC 1.6.2.2), which uses NADH as a cofactor, rises slightly at low pHs (like those developed in hypoxic areas of tumours) and can reduce other quinone-based compounds such as mitomycin C, producing the semiquinone radical under hypoxic conditions (Hodnick and Sartorelli, 1993). Cyt$b_5$R is a promising enzyme but its levels in human tumour tissues are not known.

If enzyme-directed bioreductive agents are to have a place in cancer therapy, it is essential that the levels of reductases in both tumours and normal tissues are known. Therefore, in this work we have measured DTD in two different ways, by activity and protein expression, in a number of paired human lung and breast normal and tumour tissues. The variability of DTD is analysed and the possible implications discussed. The activity of the one-electron reducing enzyme, Cyt$b_5$R, was measured for comparison and because of its possible role in the selective activation of bioreductive drugs under hypoxia (Plumb et al, 1994*b*; Robertson et al, 1994).

### MATERIALS AND METHODS

### Patient characteristics

Biopsy specimens of 19 lung solid tumours and 20 breast tumours were obtained from two hospitals in Pamplona, Spain. Primary solid tumours were obtained together with macroscopically normal tissue from the same subject. Full medical records were kept for all the subjects in the study. Records were not collected until after the tissues had been analysed for reductase levels. The following data were obtained: age, sex, ethnic origin, current pathologies and medication, tobacco use, tumour histology and grade, presence of metastasis and the tumour staging according to the TNM classification status (AJCC, 1992). Patient characteristics are briefly described in Tables 1 and 2.

The mean age of the patients with lung tumours was 65 years. All of them were smokers or ex-smokers except for the two women in the study, who had no recorded tobacco product use. In general, they were receiving concomitant medication for non-malignant pathologies, such as cardiac and/or respiratory pathologies. Previous malignant pathologies had occurred in six cases (patients 9, 15, 13, 17, 19 and 11); for the last three this was also in the lungs. Three patients had prior chemotherapy: patient 5 with cisplatin, vinorelbine and taxol; patient 18 with cisplatin, carboplatin and mitomycin C; and patient 19 with cisplatin, vinorelbine and mitomycin C. Patient 9 took tamoxifen during the last month before the operation because she suffered a parallel breast carcinoma. The tumour histological type, grade, TNM status and staging appear in Table 1.

The mean age of the patients with breast tumours was 51 years. All were females undergoing radical or conservative surgery. Previous malignant pathologies occurred in six cases (patients 1, 11, 16, 4, 14 and 19), in the breast in the last three cases. Non-malignant breast pathologies were suffered in two cases (patients 6 and 13). Patient 4 was undergoing hormone therapy with tamoxifen

 © Cancer Research Campaign 1997

**Table 2** Characteristics of breast tumour patients

| Patient | HR[a] | Histological type[b] | Grade[c] | TNM[d] | Stage[e] |
|---|---|---|---|---|---|
| 1 | ER(+)PR(+) | DIC* | G3 | T2N0M0 | IIA |
| 2 | ER(+)PR(+) | LIC | G2 | T2N1M0 | IIB |
| 3 | ER(–)PR(+) | DC | G1 | T2N0M0 | IIA |
| 4 | ER(–)PR(–) | LIC | G3 | T4N2M0 | IIIB |
| 5 | ER(+)PR(–) | DIC | G2 | T1N0M0 | I |
| 6 | ER(–)PR(+) | DIC | G3 | T1N0M0 | I |
| 7 | ER(+)PR(+) | DIC | G3 | T2N1M0 | IIB |
| 8 | ER(–)PR(–) | DIC* | G2 | T2N0M0 | IIA |
| 9 | ER(+)PR(+) | CA | G1 | T1N0M0 | I |
| 10 | ER(–)PR(+) | MixedC* | G2 | T3N1M0 | IIIA |
| 11 | ER(+)PR(–) | DIC | G1 | T1N1M0 | IIA |
| 12 | ER(–)PR(+) | DIC* | G2 | T1N0M0 | I |
| 13 | ER(+)PR(+) | DIC | G3 | T2N0M0 | IIA |
| 14 | ER(+)PR(+) | DIC* | G3 | T1N1M0 | IIA |
| 15 | ER(+)PR(+) | DIC | G3 | T2N0M0 | IIA |
| 16 | ER(+)PR(–) | IC | G2 | T2N1M1 | IV |
| 17 | ER(–)PR(–) | DIC* | G3 | T2N1M0 | IIB |
| 18 | ER(+)PR(–) | DIC | G3 | T2N0M0 | IIA |
| 19 | ER(+)PR(–) | MixedC | G2 | T2N0M0 | IIA |
| 20 | ? | DIC | G2 | T4N1M1 | IV |

[a]HR: tumour presence (+) or absence (–) of the oestrogen (ER) or progesterone (PR) receptors. ?, not registered. [b]Histological type: DIC, ductal infiltrating carcinoma; LIC, lobular infiltrating carcinoma; DC, in situ ductal carcinoma; CA, colloid adenocarcinoma; MixedC, mixed ductal and lobular infiltrating carcinoma; CI, intraductal carcinoma. *Although this was the most prevailing histological type in these samples, they presented areas or focus where other histological types were represented. [c]Grade: G1, well-differentiated; G2, moderately differentiated; G3, poorly differentiated. [d]TNM classification and [e]stage according to AJCC (1992).

because her pathology was a recurrence from a previous breast carcinoma. Although other patients received chemotherapy, only patient 20 was given it during the months before surgery (four cycles of cyclophosphamide, doxorubicin and 5-fluorouracil).

### Tissues

Tissues were obtained from the pathology laboratory. They were frozen in liquid nitrogen after arrival from the operating theatre immediately after operation, and kept at –80°C until transported in liquid nitrogen to the research laboratory, where they were stored at –135°C until processed. Tumour and normal tissues were frozen and stored separately. Batches of three or four paired tissues were processed for enzyme assays at the same time. A piece of liver from a rat, which had been previously frozen and stored in the same way as the human tissues, was introduced for processing with each batch.

The frozen tissues were pulverized with liquid nitrogen in a porcelain mortar. The powder was weighed and resuspended by several strokes of a hand-held glass homogenizer, in 3 volumes of ice-cold homogenizing buffer (potassium phosphate/potassium chloride 10 mM/0.1 M, pH = 7.8, 0.1 mM EDTA, 2 mM phenylmethyl sulphonyl fluoride (PMSF) and 80 mg $l^{-1}$ trypsin inhibitor). Finally, the samples were homogenized using an Ultra Turrax-T25 homogenizer (Janke & Kunkel, IKA-labortechnik) or a Potter homogenizer (B Brown). The temperature was maintained at 4°C throughout by working on ice. The homogenate was then centrifuged at 4°C, at 15 000 *g* for 20 min. The S9 fraction of the tissues was obtained in the supernatants. These supernatants were then aliquoted and stored at –80°C until used for enzyme activity measurements or for Western blot experiments. Reproducibility was tested with the rat liver samples. Inter- and intra-assay variation was about 10%. Protein concentration of the supernatants was determined by the bicinchoninic acid assay (Smith et al, 1985).

### Enzyme assays

DTD activity was determined spectrophotometrically by following the reduction of cytochrome c at 550 nm, using a modification of the Ernster method (Ernster, 1967), as reported in detail elsewhere (Riley and Workman, 1992). Essentially, the reactive mixture contained cytochrome c (77 µm), bovine serum albumin (BSA) (0.14%, w/v), NADH (0.2 mM) as the cofactor and menadione (20 µM) as the intermediate electron acceptor. The reaction was initiated with the addition of 10 µl of the S9 fraction of the tissues. Reactions were conducted at 37°C in a total volume of 1 ml of Tris-HCl buffer 50 mM (pH 7.4), in the presence and absence of the specific inhibitor dicoumarol (10 µM). DTD activity was expressed as the fraction of activity measured that was dicoumarol inhibitable. Cytochrome $b_5$ reductase activity was calculated as the cytochrome reductase activity measured with NADH (0.2 mM) as the cofactor, in the absence of a substrate. Both activities were expressed as nmol cytochrome c ($\varepsilon$ 21.1 × $10^3$ M $cm^{-1}$) reduced $min^{-1}$ $mg^{-1}$ protein.

### DTD expression

The level of expression of DTD in S9 tissue fractions (50 µg, total protein) was measured by Western blot analysis after sodium dodecyl sulphate-polyacrylamide gel electrophoresis (SDS-PAGE), using 12% polyacrylamide gels (Laemmli, 1970). Resolved proteins were electrotransferred to Immobilon-P membranes in transfer buffer (Tris 48 mM, glycine 39 mM, SDS 0.037% p/v, methanol 10% v/v). The blots were probed with a primary polyclonal antibody

© Cancer Research Campaign 1997 

**Table 3** Reductase activities in human normal and tumour lung tissues[a]

| | DT-diaphorase | | | | Cytochrome $b_5$ Reductase | | |
|---|---|---|---|---|---|---|---|
| Patient | Normal | Tumour | T/N[b] | S[c] | Normal | Tumour | T/N[b] |
| 1 | 184.1 ± 19.3 | 114.0 ± 4.7 | 0.6 | 21.3/20.1 | 174.5 ± 2.0 | 22.5 ± 1.0 | 0.1 |
| 2 | 7.9 ± 1.0 | 96.2 ± 4.9 | 12.2 | 4.2/nd | 40.5 ± 0.3 | 30.5 ± 0.8 | 0.7 |
| 3 | 20.6 ± 2.2 | 176.4 ± 3.3 | 8.5 | 82.5/nd | 76.1 ± 0.6 | 51.7 ± 0.0 | 0.7 |
| 4 | 7.9 ± 1.5 | 127.1 ± 6.0 | 16.0 | 24.6/nd | 49.4 ± 1.8 | 56.2 ± 1.2 | 1.1 |
| 5 | ND | 79.0 ± 3.9 | 79.0(+) | 12.4/nd | 25.8 ± 0.0 | 30.3 ± 1.6 | 1.2 |
| 6 | 26.5 ± 0.2 | 183.2 ± 3.5 | 6.9 | 46.7/4.7 | 39.9 ± 0.8 | 45.5(*) | 1.1 |
| 7 | 37.9 ± 1.7 | 92.5 ± 1.8 | 2.4 | 8.6/5.3 | 20.2 ± 0.5 | 21.7 ± 0.0 | 1.1 |
| 8 | 20.9 ± 1.0 | 232.6 ± 8.4 | 11.1 | 57.1/nd | 63.0 ± 1.0 | 11.6 ± 0.0 | 0.2 |
| 9 | 10.7 ± 0.4 | 965.8 ± 10.1 | 90.3 | 11.4/nd | 35.2 ± 0.4 | 30.1 ± 0.4 | 0.8 |
| 10 | 19.8 ± 0.2 | 434.3 ± 22.9 | 21.9 | 76.4/nd | 2.7 ± 0.4 | 17.2 ± 1.6 | 6.3 |
| 11 | 6.5 ± 0.3 | 101.6 ± 3.3 | 15.7 | 0.6/nd | 3.8 ± 0.2 | 41.5 ± 1.4 | 11.0 |
| 12 | 39.1 ± 1.6 | 32.4 ± 1.8 | 0.8 | nd/nd | 64.7 ± 0.9 | 41.1 ± 0.1 | 0.6 |
| 13 | 20.0 ± 2.6 | 19.6 ± 1.0 | 1.0 | nd/nd | 54.8 ± 7.2 | 32.9 ± 0.3 | 0.6 |
| 14 | 5.2 ± 1.9 | 13.0 ± 2.2 | 2.5 | nd/nd | 64.5 ± 1.1 | 46.1 ± 0.1 | 0.7 |
| 15 | 12.3 ± 0.6 | 1.8 ± 0.2 | 0.1 | nd/nd | 64.2 ± 1.9 | 3.7 ± 0.3 | 0.0 |
| 16 | 2.8 ± 0.4 | 1.0 ± 0.3 | 0.3 | nd/1.5 | 29.6 ± 1.3 | 25.6 ± 1.3 | 0.9 |
| 17 | ND | 1.1 ± 0.4 | 1.1(+) | nd/nd | 15.9 ± 0.4 | 9.3 ± 0.1 | 0.6 |
| 18 | 6.1 ± 2.2 | 1.3 ± 0.1 | 0.2 | nd/nd | 84.0 ± 0.1 | 22.4 ± 0.2 | 0.3 |
| 19 | 3.3 ± 0.0 | 4.2 ± 0.6 | 1.3 | nd/nd | 25.7 ± 0.1 | 38.1 ± 1.8 | 1.5 |

[a]DT-diaphorase and $Cytb_5R$ activities were measured in the S9 fractions from paired human lung tumour and normal tissue, as described in the text. Reduction rates are mean ± s.e.m. of three assays of a given homogenate, expressed in nmol cytochrome c reduced $min^{-1}$ $mg^{-1}$ protein. (*), Only one experiment could be carried out. [b]T/N, ratio between tumour and normal tissue activity. ND, not detectable, activity below 1 nmol $min^{-1}$ $mg^{-1}$ protein. (+), it is only an estimation because N is not known. [c]S, results of the densitometric scans in tumour sample/normal sample. nd, not detected.

raised against purified rat DTD, and developed with a protein A–horseradish peroxidase conjugate and the chemical reagents of an enhanced chemoluminescence commercial kit (Amersham). Films were exposed for no more than 20 min. The optical density × mm of the bands was determined by scanning densitometry (Image Master 1D, Pharmacia Biotech). The results were normalized with an internal control (cell extracts from a human lung tumour cell line: HTB-54), and afterward expressed as a percentage.

### Statistical analysis

The Mann–Whitney *U*-rank sum test was used for assessing differences between tumour and normal tissues and between tumour tissues from different patients. Correlation between DTD activity and DTD protein expression was determined by the non-parametric Spearman correlation test. A probability of $P < 0.05$ was considered significant in all cases.

### RESULTS

The histological type of the lung tumours analysed are presented in Table 1. All were non-small-cell lung cancers (NSCLC): ten epidermoid carcinomas, seven adenocarcinomas, one mucoepidermoid carcinoma and one carcinoid tumour. The distribution of cytosolic DTD activities in the tumour and paired normal tissue of each patient is shown in Table 3. We found low values for this enzyme in almost all samples of normal tissue assayed. The exception was patient 1, in whose normal tissue we also found an elevated value for $Cytb_5R$ activity. Excluding this value, the normal lung activity ranged between 0 and 39 nmol $min^{-1}$ $mg^{-1}$ protein (mean 15.59 ± 2.63). For the tumour tissue, we could distinguish two different groups: in patients 1–11, DTD was high, ranging between 79 and 965 nmol $min^{-1}$ $mg^{-1}$ protein and, with the exception of patient 1, it was increased with respect to the normal tissue ($P < 0.001$) by a factor of 2.4–90.3. In patients 12–19, tumour DTD activity was as low as in the normal tissue.

We investigated the heterogeneity in enzyme activity, analysing the influence of different factors in tumour DTD. We could not find any difference between tumour activity from patients with different habits of tobacco use or from subjects receiving prophylactic anti-coagulant therapy (which is believed to inhibit the enzyme). When we compared tumour DTD activity with the tumour staging and with histological grade or type, we found significant differences ($P < 0.05$) in all cases (data shown in Table 4).

The DTD activity in the 20 different sets of paired breast tissues is presented in Table 5. In 70% of the tumour samples (2–15), DTD activity was over 80 nmol $min^{-1}$ $mg^{-1}$ protein (range 83–267 nmol $min^{-1}$ $mg^{-1}$ protein). The remaining 30% of the tumour samples showed low levels of activity, ranging between 5 and 42 nmol $min^{-1}$ $mg^{-1}$ protein. In the normal tissue, DTD activity was low in the majority of the samples (6–20) ranging between 2 and 45 nmol $min^{-1}$ $mg^{-1}$ protein. The remaining five samples (1–5) showed elevated levels of DTD activity ranging between 62 and 342 nmol $min^{-1}$ $mg^{-1}$ protein. Analysing the paired samples showed that DTD activity was generally induced in tumours ($P < 0.001$). The influence of several factors that could account for the heterogeneity in tumour DTD activity was analysed. We only found significant differences when we compared tumoral DTD activity from patients positive or negative for the oestrogen receptor. DTD activity was higher in tumours that were negative for this receptor (range 28–267 nmol $min^{-1}$ $mg^{-1}$ protein) than in tumours that were ER positive (range 34–159 nmol $min^{-1}$ $mg^{-1}$ protein). No other factors, such as progesterone receptor presence, tumour stage, grade or histological type, gave a significant correlation with DTD activity.

© Cancer Research Campaign 1997

**Table 4** Different factors affecting tumour DTD activity.

| Factor | | DTD[a] | Range | n[b] |
|---|---|---|---|---|
| Grade | G1* | 179.8 ± 37.3 | 127–232 | 2 |
| | G2 + G3 | 48.6 ± 15.8 | 0.96–176 | 13 |
| Stage | I + II* | 108.5 ± 22.6 | 1–232 | 10 |
| | IIIA + IIIB | 22.4 ± 15.8 | 1–92 | 5 |
| Histological type | AD* | 140.4 ± 14.8 | 101–183 | 5 |
| | EC | 57.2 ± 28.7 | 0–232 | 8 |

[a]DTD, DT-diaphorase activity (mean ± s.e.m.). Units are nmol min$^{-1}$ mg$^{-1}$ protein. Samples 9 and 10 were excluded from the analysis. [b]*n*, Number of individual samples (biopsy specimen). *Tumour DTD activity significantly higher ($P < 0.05$). The Mann–Whitney *U*-test was used for statistical analysis.

**Table 5** Reductase activities in human normal and tumour breast tissues[a]

| Patient | DT-diaphorase | | | | Cytochrome $b_5$ reductase | | |
|---|---|---|---|---|---|---|---|
| | Normal | Tumour | T/N[b] | S[c] | Normal | Tumour | T/N[b] |
| 1 | 137.3 ± 8.3 | 41.8 ± 2.7 | 0.3 | 4.3/68.7 | 69.7 ± 0.0[a] | 42.4 ± 0.2 | 0.6 |
| 2 | 258.6 ± 1.3 | 99.0 ± 25.9 | 0.4 | 21.5/56.6 | 24.4 ± 0.0 | NS | – |
| 3 | 342.4 ± 1.2 | 267.3 ± 11.0 | 0.8 | 38.6/41.5 | 23.1 ± 0.1 | 16.9 ± 0.0 | 0.7 |
| 4 | 93.1 ± 2.6 | 157.9 ± 5.7 | 2.5 | 39.1/0.6 | 10.9 ± 0.1 | 9.6 ± 0.7 | 0.9 |
| 5 | 62.0 ± 9.8 | 159.5 ± 2.5 | 2.6 | 31.9/9.5 | 10.67* | 11.9 ± 0.7 | 1.1 |
| 6 | 44.7 ± 3.1 | 130.7 ± 3.8 | 2.9 | 11.7/3.1 | 28.7 ± 0.6 | 15.9 ± 3.8 | 0.5 |
| 7 | 6.0 ± 0.7 | 96.2 ± 4.5 | 16.0 | 5.8/1.7 | 4.8 ± 0.2 | 17.6 ± 0.4 | 3.6 |
| 8 | 10.6 ± 1.7 | 197.7 ± 3.0 | 18.6 | 50.4/nd | 38.3 ± 0.8 | 30.7 ± 0.9 | 0.8 |
| 9 | 3.4 ± 0.3 | 82.9 ± 3.7 | 24.4 | 23.5/nd | 13.0 ± 0.3 | 19.7 ± 0.6 | 1.5 |
| 10 | 17.8 ± 0.9 | 119.5 ± 2.4 | 6.7 | 53.8/7.9 | 17.3 ± 0.4 | 14.9 ± 0.3 | 1.1 |
| 11 | 12.7 ± 1.1 | 122.9 ± 3.5 | 9.7 | 53.5/nd | – | – | – |
| 12 | 26.4 ± 2.7 | 177.1 ± 1.4 | 6.7 | nd/nd | – | – | – |
| 13 | 9.6 ± 0.9 | 129.8 ± 3.9 | 13.5 | 86.5/nd | 9.4 ± 0.4 | 14.9 ± 0.3 | 1.7 |
| 14 | 23.0 ± 1.1 | 127.0 ± 5.0 | 5.5 | 3.9/nd | 11.4 ± 0.9 | 8.8 ± 1.3 | 1.8 |
| 15 | 2.3 ± 0.5 | 96.9 ± 7.8 | 42.1 | 24.2/nd | NS | 8.9 ± 0.4 | – |
| 16 | 2.3 ± 0.0 | 33.9 ± 0.8 | 14.7 | 2.7/nd | 4.7 ± 0.3 | 7.9 ± 0.4 | 1.7 |
| 17 | 11.6 ± 0.4 | 27.6 ± 1.2 | 2.4 | 23.1/0.5 | 12.1 ± 0.4 | 22.4 ± 0.5 | 1.8 |
| 18 | 4.4 ± 0.8 | 34.5 ± 2.0 | 7.8 | 3.6/nd | 23.2 ± 0.9 | 15.9 ± 0.7 | 0.7 |
| 19 | 3.3 ± 0.0 | 5.3 ± 0.7 | 1.6 | nd/nd | 7.23 ± 0.7 | 4.55* | 0.6 |
| 20 | 14.3 ± 1.6 | 11.2 ± 1.3 | 0.8 | nd/nd | 12.6 ± 0.1 | 7.9 ± 0.7 | 0.6 |

[a]DT-diaphorase and Cytb$_5$R activities were measured in the S9 fractions from paired human breast tumour and normal tissue, as described in the text. Reduction rates are mean ± s.e.m. of three assays of a given homogenate, expressed in nmol cytochrome c reduced min$^{-1}$ mg$^{-1}$ protein. (*) Only one experiment could be carried out. [b]T/N, ratio between tumour and normal tissue activity. [c]S, results of the densitometric scans in tumour sample/normal sample. nd, not detected.

Figures 1 and 2 show several representative examples of the Western blot experiments for determining DTD protein expression in the samples. The correlation of DTD activity vs DTD expression measured densitometrically was significant for both tumour and normal tissue. The results of the densitometric scanning, expressed as a percentage referred to the internal control, are included in Tables 3 and 5. Spearman correlation coefficient was $r_s = 0.753$*** ($P < 0.001$) for breast normal tissue and $r_s = 0.532$* ($P < 0.05$) for breast tumour tissue; $r_s = 0.924$*** ($P < 0.001$) for lung tumour tissue (excluding samples 9 and 3) and $r_s = 0.651$** ($P < 0.01$) for normal tissue.

We measured the activity of the one-electron reducing enzyme Cytb$_5$R in paired tumour and normal tissues (Tables 3 and 5). We found less heterogeneity in this enzyme activity than that detected for DTD activity. In normal lung tissue, Cytb$_5$R varied between 3 and 174 nmol min$^{-1}$ mg$^{-1}$ protein; patient 1 exhibited a very high value (174 ± 2 nmol min$^{-1}$ mg$^{-1}$ protein) and patients 10 and 11 showed a very low value (2.7 and 3.8 respectively); excluding these values, the mean is 48.00 ± 4.85 nmol min$^{-1}$ mg$^{-1}$ protein. In the lung tumour tissue, the activity was in general lower; it ranged between 3 and 56 nmol min$^{-1}$ mg$^{-1}$ protein (mean 30.42 ± 3.24 nmol min$^{-1}$ mg$^{-1}$ protein). The Mann–Whitney *U*-rank sum test revealed significant differences between normal and tumour tissue ($P < 0.05$). Normal breast tissue Cytb$_5$R activity ranged between 0 and 69 nmol min$^{-1}$ mg$^{-1}$ protein, while tumour Cytb$_5$R activity ranged between 8 and 42 nmol min$^{-1}$ mg$^{-1}$ protein (Table 5). In 56% of the patients, Cytb$_5$R in tumour tissue was lower than in the normal tissue, but the difference between the activities in the two types of tissue was not significant.

### DISCUSSION

In the present work, we have distinguished two different behaviours concerning the two-electron reducing enzyme DTD. The activity of this enzyme was induced significantly in tumour tissue from 70% of the breast cancer patients and from 53% of the lung

© Cancer Research Campaign 1997 

**Figure 1** Western blot examples of human lung tissue S9 fractions (**A** and **B**). Total protein (50 μg) was developed with polyclonal anti-rat DTD. Lanes 1, 3 and 5 contain lung tumour tissue (T), and lanes 2, 4 and 6 contain the corresponding normal tissue (N). Numbers before T or N indicate the patient number. Lane 7, internal control (C), human lung cancer cell line HTB-54 (50 μg). Lane 8, rat liver (RL) S9 fraction (50 μg)

**Figure 2** Western blot examples of human breast tissue S9 fractions (**A – C**). Total protein (50 μg) was developed with a polyclonal anti-rat DTD antibody. Tumour tissue (T), normal tissue (N) or peripheral tissue (P) are preceded by the patient number. DTD, purified rat DTD protein (10 μg). C, internal control, human lung cancer cell line HTB-54 (50 μg). RL, rat liver S9 fraction (50 μg). The approximate molecular weight of the band detected is indicated cancer patients included in this study, confirming some previous observations (Schlager and Powis, 1990; Smitskamp-Wilms et al, 1995). However, there were a considerable number of exceptions to this behaviour: lung tumours from patients 12–19 and breast tumours from patients 1 and 16–20 showed low DTD activity.

Studies on the regulation of expression of DTD (Prochaska and Talalay, 1988; Cresteil and Jaiswal, 1991; Belinsky and Jaiswal, 1993) indicated that DTD can be induced by cytotoxic drugs. Four patients in our study received prior chemotherapy, and there was no evidence of induction of DTD levels in the tumours of treated patients. In fact, three of them presented very low values (patients 18 and 19 from Table 3 and patient 20 from Table 5), and the other patient presented levels that were not especially high (patient 5 from Table 3).

We could not find any difference between the tumour DTD activity of subjects who were past smokers and subjects who were active smokers, since patients who were current smokers (see Table 1) presented a considerable increase in their tumour DTD activity. Our data are in disagreement with those published previously. Schlager and Powis (1990) described the cigarette smoking history of the subjects as a major cause of variability of DTD activity. They found that lung tumours from non-smokers or past smokers exhibited considerably higher DTD activity in comparison with the corresponding normal tissue, but tumours from smokers showed no increase in tumour DTD.

We found that several tumour characteristics correlated with DTD tumour activity: grade, stage and histological type. DTD activity seemed to be more induced in well-differentiated tumours than in the moderately or poorly differentiated ones. It also tended to be higher in tumours in stage I or II and in epidermoid carcinomas.

A higher expression of *DTD* gene (NQO1 locus) in normal tissue surrounding hepatic tumours in comparison with the normal tissues from the same origin has been reported (Cresteil and Jaiswal, 1991). This fact and other studies suggested that soluble factor(s) from tumour cells could diffuse into the surrounding normal tissues and activate the expression of NQO1 by an unknown mechanism (Belinsky and Jaiswal, 1993). If this were true, the high values of the enzyme, both activity and protein expression in the normal tissue from some patients, could be explained. Also, the possibility of tumoral infiltration cannot be discarded.

There was an absence of correlation between DTD activity and DTD expression in lung tumour samples 9 and 3. Other enzymes can use menadione as a substrate: carbonyl reductase and $Cytb_5R$ are examples; and both of these can be inhibited by dicoumarol, the DTD inhibitor (Wermuth, 1981; Hodnick and Sartorelli, 1993). Therefore, it could be that other enzymes in the tumour tissue of patient 9 contributed to the apparently elevated DTD activity. In tumour tissue from patient 3, higher protein expression than activity was measured. There is little information available on substrate specificities of the different forms of NQOs; the NQO2-encoded protein is 43 amino acids shorter at the carboxy terminal end, and several authors have suggested distinct affinities of diaphorases (NQOs) for the metabolism of several substrates, including menadione (Edwards et al, 1980; Segura-Aguilar and Lind, 1987). The NQO2 cDNA-encoded protein was found to be 50-fold to 100-fold less active at reducing the menadione readily metabolized by NQO1 protein (Jaiswal et al, 1990), so we cannot discard the theory that patient 3 had this isoenzyme.

Regarding the one-electron reducing enzyme $Cytb_5R$, we found no important differences between normal and tumour tissues. The levels of activity found in tumours were as much as 16 times lower than those found for DTD, which is consistent with previous results in lung tumour cell lines (Plumb et al, 1994*b*). The lack of induction of $Cytb_5R$ makes it clear that different regulatory mechanisms are implicated in the expression of the two enzymes. In consequence, these results do not encourage a therapeutic strategy based on this reductase.

In our opinion, this study questions the expectations created around the enzyme DTD. Although it is true that there are clear differences of expression in tumour vs normal tissue in some patients, it is also true that the level of induction is very heterogeneous and seldom tenfold higher. In several in vitro studies, a significant correlation has been obtained between aerobic sensitivity to EO9 and DTD activity, but in these studies the range of DTD activity among the different cell lines used was much higher than that found in human tumours. This fact, together with the high proportion of exceptions encountered in the tumour tissue of a number of patients, provides a note of caution. In short, DTD-mediated chemotherapy should be indicated only for individual patients with a demonstrated, very high level of activity of this particular enzyme measured in tumour biopsy specimens.

 © Cancer Research Campaign 1997

In summary, this article provides more data on bioreductive enzyme levels found in clinical samples and encourages us to study a greater number of human tumours in order to determine the true potential for therapeutic exploitation of DTD-dependent bioreductive drugs.

### ACKNOWLEDGEMENT

This research was supported by Zeneca, within the National Plan of Scientific and Technological Investigation of Spain.

### REFERENCES

American Joint Committee on Cancer (AJCC) (1992) *Manual for Staging of Cancer*, 4th edn. Beahrs OH, Henson DE, Hutter RVP, Kennedy BJ (eds). J.B. Lippincott Company: Philadelphia

Belinsky M and Jaiswal AK (1993) NAD(P)H: quinone oxidoreductase (DT-diaphorase) expression in normal and tumor tissues. *Cancer Metast Rev* **12**: 103–117

Chesis PL, Levin DE, Smith MT, Ernster L and Ames BN (1984) Mutagenicity of quinones: pathways of metabolic activation and detoxification. *Proc Natl Acad Sci USA* **81**: 1696–1700

Cresteil T and Jaiswal AK (1991) High levels of expression of the NAD(P)H: quinone oxidoreductase (NQO1) gene in tumor cells compared to normal cells of the same origin. *Biochem Pharmacol* **42**: 1021–1027

Doherty N, Hancock SL, Kaye S, Coleman CN, Shulman L, Marquez C, Mariscal C, Rampling R, Senan S and Roemeling RV (1994) Muscle cramping in phase I clinical trials of tirapazamine (SR4233) with and without radiation. *Int J Radiat Oncol Biol Phys* **29**: 379–382

Edwards Y, Potter J and Hopkinson DA (1980) Human FAD-dependent NAD(P)H diaphorase. *Biochem J* **187**: 429–436

Ernster L (1967) DT-diaphorase. *Methods Enzymol* **10**: 309–317

Fitzsimmons SA, Workman P, Grever M, Kenneth P, Camalier R and Lewis AD (1996) Reductase enzyme expression across the National Cancer Institute Tumor Cell Line Panel: correlation with sensitivity to mitomycin C and E09. *J Natl Cancer Inst* **88**: 259–269

Hodnick WF and Sartorelli AC (1993) Reductive activation of mitomycin C by NADH-Cytb$_5$R reductase. *Cancer Res* **53**: 4907–4912

Iyanagi T and Yamazaki I (1969) One-electron-transfer reactions in biochemical systems. III. One-electron reduction of quinones by microsomal flavin enzymes. *Biochim Biophys Acta* **172**: 370–381

Jaiswal A, Burnett P, Adesnik M and McBride OW (1990) Nucleotide and deduced aminoacid sequence of human cDNA (NQO2) corresponding to a second member of the polymorphism at the NQO2 gene locus on chromosome 6. *Biochemistry* **29**: 1899–1906

Kennedy KA, Teicher BA, Rockwell S and Sartorelli AC (1980) The hypoxic tumor cell: a target for selective cancer chemotherapy. *Biochem Pharmacol* **29**: 1–8

Laemmli U-K (1970) Cleavage of structural proteins during the assembly of head of bacteriophage T4. *Nature* **227**: 680–685

Lewis AD, Holyoake TL, Dunlop DJ, Pragnell I and Workman P (1993) Lack of myelosuppression with low DT-diaphorase. *Proc Am Assoc Cancer Res* **34**: A2057

Lind C (1985) Relationship between the role of reduction of benzo(a)pyrene-3,6-quinone and the formation of benzo(a)pyrene-3,6-quinol glucuronides in rat liver microsomes. *Biochem Pharmacol* **34**: 895–897

Nohl H, Jordan W and Youngman RJ (1986) Quinones in biology, functions in electron transfer and oxygen activation. *Adv Free Rad Biol Med* **2**: 211–279

Plumb JA, Gerritsen M and Workman P (1994*a*) DT-diaphorase protects cells from the hypoxic cytotoxicity of indoloquinone EO9. *Br J Cancer* **70**: 1136–1143

Plumb JA, Gerritsen M, Milroy R, Thomson P and Workman P (1994*b*) Relative importance of DT-diaphorase and hypoxia in the bioactivation of E09 by human lung tumour cells lines. *Int J Radiat Oncol Biol Phys* **29**: 295–299

Prochaska HJ and Talalay P (1988) Regulatory mechanisms of monofunctional and bifunctional anticarcinogenic enzyme inducers in murine livers. *Cancer Res* **48**: 4776–4782

Prochaska HJ, Talalay P and Sies H (1987) Direct protective effect of NAD(P)H: quinone reductase against menadione-induced chemiluminescence of postmitochondrial fractions of mouse liver. *J Biol Chem* **262**: 1931–1934

Riley RJ and Workman P (1992) DT-diaphorase and cancer chemotherapy. *Biochem Pharmacol* **43**: 1657–1659

Robertson N, Haigh A, Adams GE and Stratford IJ (1994) Factors affecting the sensitivity of EO9 in rodent and human tumour cells in vitro: DT-diaphorase and hypoxia. *Eur J Cancer* **30A**: 1013–1019

Robinson C and Castañer J (1995) Tirapazamine. Bioreductive hypoxic cell cytotoxin. *Drugs of the Future* **20**: 256–263

Sartorelli AC (1988) Therapeutic attack of hypoxic cells of solid tumors: presidential address. *Cancer Res* **48**: 775–778

Schellens JH, Planting AS, Van Acker BA, Loos WJ, De-Boer Dennert M, Van Der Burg ME, Koier I, Krediet RT, Stoter G and Verwey J (1994) Phase I and pharmacologic study of the novel indoloquinone bioreductive alkylating cytotoxic drug EO9. *J Natl Cancer Inst* **86**: 906–12

Schlager JJ and Powis G (1990) Cytosolic NAD(P)H: (quinone acceptor) oxidoreductase in human normal and tumor tissue: effects of cigarette smoking and alcohol. *Int J Cancer* **45**: 403–409

Schor NA and Cornelisse CJ (1983) Biochemical and quantitative histochemical study of reduced pyridine nucleotide dehydrogenation by human colon carcinomas. *Cancer Res* **43**: 4850–4855

Segura-Aguilar JE and Lind C (1987) Isolation and characterization of DT-diaphorase enzymes from rat liver. *Chem Scripta* **27A**: 37–41

Sies H (1986) Biochemistry of oxidative stress. *Angew Chem Int Ed Eng* **25**: 1058–1071

Smith PK, Krohn IR, Hermanson GT, Mallia AK, Gartner FH, Provenzano MD, Fujimoto EK, Goeke NM, Olson BJ and Klenk DC (1985) Measurement of protein using bicinchoninic acid. *Anal Biochem* **150**: 76–85

Smitskamp-Wilms E, Giaccone G, Pinedo HM, Van Der Laan BFAM and Peters GJ (1995) DT-diaphorase activity in normal and neoplastic human tissues; an indicator for sensitivity to bioreductive agents? *Br J Cancer* **72**: 917–921

Suttle JN (1985) Vitamin K-dependent carboxylase. *Annu Rev Biochem* **54**: 459–477

Tomasz M, Lipman R, Chowdary D, Pawlek J, Verdine GL and Nakanishi K (1987) Isolation and structure of covalent cross-link adduct between mitomycin C and DNA. *Science* **235**: 1204–1208

Wermuth B (1981) Purification and properties of an NADPH-dependent carbonyl reductase from human brain. Relationship to prostaglandin 9-ketoreductase and xenobiotic ketone reductase. *J Biol Chem* **256**: 1206–1213

Workman P (1992) Keynote address: bioreductive mechanisms. *Int J Radiat Oncol Biol Phys* **22**: 631–637

Workman P (1994) Enzyme-directed bioreductive drug development revisited: a commentary on recent progress and future prospects with emphasis on quinone anticancer agents and quinone metabolising enzymes, particularly DT-diaphorase. *Oncol Res* **6**: 461–475

Workman P and Stratford IJ (1993) The experimental development of bioreductive drugs and their role in cancer therapy. *Cancer Metast Rev* **12**: 73–82

Workman P and Walton MI (1990) Enzyme-directed bioreductive drug development. In *Selective Activation of Drugs by Redox Processes*, Adams GE, Breccia A, Fielden EM and Wardman P (eds), pp. 173–191. Plenum Press: New York

© Cancer Research Campaign 1997